# Can vaginal lactobacillus suppositories help reduce urinary tract infections?

**DOI:** 10.1007/s00192-023-05568-4

**Published:** 2023-07-01

**Authors:** Poone S. Shoureshi, Clarissa Niino, Karyn S. Eilber

**Affiliations:** 1 Department of Urology, Cedars-Sinai Medical Center, Los Angeles, CA USA; 2 Department of Obstetrics & Gynecology, Cedars-Sinai Medical Center, Los Angeles, CA USA

**Keywords:** Vaginal probiotic, Vaginal supplement, Lactobacillus, Recurrent urinary tract infections, Vaginal health

## Abstract

**Introduction and hypothesis:**

Recurrent urinary tract infections (rUTIs) are a burden to patients and the health care economy. Vaginal probiotics and supplements have gained significant attention in mainstream media and lay press as a non-antibiotic alternative. We performed a systematic review to determine whether vaginal probiotics are an effective means of prophylaxis for rUTI.

**Methods:**

A PubMed/MEDLINE article search was performed from inception to August 2022 for prospective, in vivo use of vaginal suppositories for the prevention of rUTIs. Search terms included: vaginal probiotic suppository (34 results), vaginal probiotic randomized (184 results), vaginal probiotic prevention (441 results), vaginal probiotic UTI (21 results), and vaginal probiotic urinary tract infection (91 results). A total of 771 article titles and abstracts were screened.

**Results:**

A total of 8 articles fit the inclusion criteria and were reviewed and summarized. Four were randomized controlled trials, with 3 of the studies having a placebo arm. Three were prospective cohort studies, and 1 was a single arm, open label trial. Five of the 7 articles that specifically evaluated for rUTI reduction with vaginal suppositories did find a decreased incidence with probiotic use; however, only 2 had statistically significant results. Both of these were studies of *Lactobacillus crispatus* and were not randomized. Three studies demonstrated the efficacy and safety of Lactobacillus as a vaginal suppository.

**Conclusion:**

Current data support the use of vaginal suppositories containing *Lactobacillus* as a safe, non-antibiotic measure, but actual reduction of rUTI in susceptible women remains inconclusive. The appropriate dosing and duration of therapy remain unknown.

## Introduction

Recurrent urinary tract infections (rUTIs) are a burden to patients and the health care economy. By definition, UTIs are considered recurrent if there are more than two in a 6-month period or three in 1 year [1]. Recurrent urinary tract infections not only impair quality of life for patients but they also have significant economic consequences [2, 3]. In 2003, the estimated health care cost of UTIs annually was US $1.6 billion, and the health care burden is certainly higher two decades later [2]. The cost of an acute UTI workup ranges from US $390 to US $730, not including the price of antibiotic treatment [4].

Women are often given multiple rounds of antibiotics between primary care physicians and specialists to treat infections. Consequently, these patients often seek non-antibiotic alternatives, such as vaginal probiotics and supplements, as these have gained significant attention in mainstream media and the lay press [5]. There is an increasing body of literature supporting the use of vaginal *Lactobacillus* suppositories to reduce candidiasis and bacterial vaginosis infections; however, the role of vaginal probiotics for the prevention of rUTI is less clear [6–9]. There is increasing data that the vaginal microbiome varies between individuals that are susceptible to urinary infections. As such, it stands to reason that a vaginal probiotic would be more effective in preventing rUTI.

The objective of this study was to determine whether vaginal probiotics are an effective means of prophylaxis for women suffering from rUTI.

## Materials and methods

This review was Institutional Review Board exempt. A systematic review was performed using the PubMed/MEDLINE database from inception to August 2022 for all prospective, in vivo studies evaluating the use of vaginal suppositories for the prevention of rUTI. Search terms included vaginal probiotic suppository (34 results), vaginal probiotic randomized (184), vaginal probiotic prevention (441 results), vaginal probiotic UTI (21 results), and vaginal probiotic urinary tract infection (91 results). Studies were excluded if they utilized oral probiotics or intravesical instillations. We also did not include studies that evaluated probiotics for the prevention of vaginal bacterial vaginosis and vulvovaginal candidiasis infections. A secondary review was also performed of the reference lists of relevant articles.

A total of 771 article titles and abstracts were screened (including duplicates), with a total of 9 articles identified. Seven were found within the search and 2 were found as references in other articles. Eight were complete articles and 1 was an abstract; the latter was excluded. The final eight articles were then carefully reviewed evaluating for patient characteristics, study intervention, presence of a control arm, type of probiotic utilized, duration of follow-up, ultimate study results, and statistical significance. Two researchers (PS and CN) independently reviewed the article content for appropriateness.

## Results

A total of 8 articles were reviewed and the results are summarized in Table 1. With regard to study design, 4 studies were randomized controlled trials, and of those, 3 had a placebo arm. Three studies were prospective cohort studies, and 1 was a single-arm, open-label trial. A total of 393 female patients were evaluated in this review. A combination of pre- and post-menopausal women were included. All study participants were adult females ≥18 years of age, except for 1, who was a 13-year-old girl.

**Table 1:** Summary of results

Study reference	Type of study	Number of patients	Inclusion criteria	Type of probiotic (dose, CFU)	Intervention	Results/conclusion	Follow up	RR (95% CI), *p* value
Baerheim et al. [10]	Randomized, double blind, placebo controlled trial	48	Premenopausal adult women with ≥ 3 UTIs in last 12 months and no UTI at study entry.	L. casei v rhamnosus (7.5 × 10^8^)	Twice weekly application for 26 weeks	No reduction in rUTI	Duration of therapy	RR 0.88 (0.48–1.62), *p* = 0.9057
Bruce and Reid [11]	Prospective cohort study	6	Females with recurrent UTIs	L. casei v rhamnosus GR-1, (1 × 10^11^)	Twice weekly intravaginal instillation for up to 5 weeks	4 week to 6 month infection free period after instillation	2–6 months	RR 0.45 (0.15–1.40), *p* = 0.2682
Cianci et al. [12]	Prospective cohort study	124	Adult women with recent BV (< 15 days) and history of recurrent vaginal infections (2 in last 12 months); and/or recent UTI (≥ 2 in last 12 months); and/or treatment with systemic antibiotics for bacterial URI in last week.	L. plantarum, (1 × 10^8^)	1 vaginal capsule daily for 6 days, then weekly for 16 weeks	No reduction in rUTI	Duration of therapy	RR not listed, *p* = 0.43 (UTI), p = 0.1 (vaginal infection)
Czaja et al. [13]	Randomized, double blind, placebo controlled trial	30	Premenopausal, adult women with ≥ 3 UTIs in prior 12 months or ≥ 2 UTIs in prior 6 months.	L. crispatus, (5 × 10^8^)	Vaginal insertion (drug or placebo) daily for 5 days	L. crispatus can be administered with minimal side effects	1 week, 4 weeks in person > 6 months telephone	RR 5.0 (0.26–96.13), *p* = 0.4642
Reid et al. [14]	Randomized, double blind (two intervention arms)	31	Premenopausal, adult women recently treated for acute UTI prior to intervention.	L. casei v rhamnosus GR-1 and L. fermentum, (1.6 × 10^9^)	Lactobacillus or placebo (skim milk powder) twice weekly for 2 weeks, then at the end of the next 2 months	Reduction in rUTI	3 months	RR 0.45 (.15–1.40), *p* = 0.2682
Sadahira et al. [15]	Single arm open label phase II clinical trial	21	Age > 20 with ≥ 2 episodes of cystitis requiring antibiotics in the prior year and current acute cysitis or acute exacerbation of chronic complicated cystitis	L. crispatus, (1 × 10^8^)	Vaginal suppository insertion every 2 days or 3 times per week for 1 year	Reduction in rUTI	1 year	No control group for RR, *p = 0.00007 (during treatment) and *p* = 0.00054 (after treatment)
Stapleton et al. [16]	Randomized, double blind, placebo controlled trial	100	Age 18–40 with current symptomatic uncomplicated cystitis and history of 1 prior symptomatic UTI treated within last 12 months	L. crispatus, (1 × 10^8^)	Vaginal insertion (drug or placebo) daily for 5 days, then weekly for 10 weeks	Reduction in rUTI	Duration of therapy	RR = 0.54 (.24–1.23), *p* = 0.2089
Uehara et al. [17]	Prospective cohort study	9	> 2 episodes of UTI in the last 12 months with history of recurrent UTI for ≥ 2 years. 2 patients with neurogenic bladder performing CIC included.	L. crispatus, (1 × 10^8^)	Vaginal suppository every 2 days for 1 year	Reduction in rUTI	Duration of therapy	No control group for RR, *p = 0.0007

*CIC* clean intermittent catheterization, *UTI* urinary tract infection, *BV* bacterial vaginosis, *URI* upper respiratory tract infection, *OAB* overactive bladder, *PVR* postvoid residual, *CFU* colony forming unit

*Significant result

**Fig. 1:**
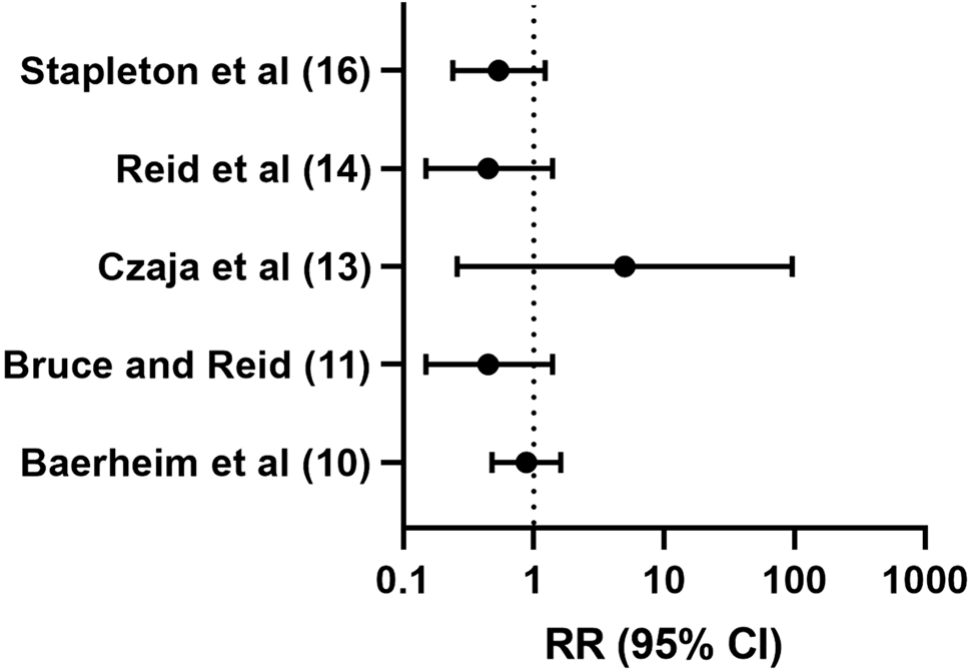
Forest plot

All of the studies utilized *Lactobacillus* as the probiotic. There were no other active ingredients. The *Lactobacillus* strains that were reviewed included *L. casei v. rhamnosus*, *L. plantarum*, *L. fermentum*, and *L. crispatus*. Specific dosages and *Lactobacillus* combinations varied between studies and are listed in Table 1. All of the dosages were at least 1 × 10^8^ colony-forming units (CFU), ranging from 1 × 10^8^ to 1 × 10^11^ CFU. Of the *Lactobacillus* genus, the most commonly studied species was *L. crispatus* (4 articles) followed by *L. casei* (3 articles). Three studies demonstrated the efficacy and safety of utilizing *Lactobacillus* in general as a vaginal suppository. No safety concerns were reported. Five of the 7 articles that specifically evaluated the use of a *Lactobacillus* suppository for rUTI reduction did find a decreased incidence with probiotic use; however, only 2 studies had statistically significant results, and both of these were studies using *L. crispatus*. A forest plot of the studies that included a risk ratio (5 of 8) is illustrated in Fig. 1.

## Discussion

Although current literature supports the use of vaginal suppositories containing *Lactobacillus* as a non-antibiotic measure to decrease rUTI in susceptible women, the available data are limited. Based on published results in two prospective studies, significant findings were associated with the utilization of *Lactobacillus crispatus* for rUTI reduction for 1 year. Most importantly, there were no reported serious adverse effects from using vaginal probiotics.

Antibiotic stewardship is vitally important in women who suffer from rUTI as they are at a high risk of developing multidrug-resistant infections. Both patients and clinicians are interested in non-antibiotic alternative treatments to avoid long-term resistance [18]. Prior focus groups and interviews of women who suffer from UTIs have shown that patients are reluctant to frequently use antibiotics and are interested in non-antibiotic alternatives [18, 19].

Our review of the current literature is important and timely for two main reasons:

1. Antibiotic resistance is an ongoing concern and repeated use of antibiotics can have untoward side effects, including gut dysbiosis and other conditions.
2. The media continues to highlight the negative side of the pharmaceutical industry and thus there is a new distrust of prescription medication in parallel with growing interest in “natural” treatments.

As clinicians, it is vital that we are familiar with the science, or lack of, behind supplements that our patients are using and that we may even recommend.

An important finding that our review revealed was that no vaginal probiotic, other than *Lactobacillus*, was identified in the literature search. Presumably this is because the vaginal microbiota is formed by a host of aerobic and anaerobic organisms, with the most prevalent being *Lactobacillus* species [20]. Studies have shown that *Lactobacillus* organisms dominate the vaginal microbiota, to help to maintain vaginal eubiosis, and most common are *L. crispatus* and *L. iners* [7, 21]. *L. crispatus* produces the most lactic acid and therefore acidifies the vaginal microbiome the most [7]. Therefore, it seems intuitive that the two studies with statistically significant results used *L. crispatus* as the main compound. Thus, if patients inquire about a vaginal probiotic for rUTI, a probiotic containing *L. crispatus* should be recommended. Unfortunately, none of the studies explained why they chose specific organism concentrations (i.e., 1 × 10^8^ CFU) and dosing regimens. Likely the dosages of probiotic used were based on the fact that lactobacilli are present at levels of 10^7^–10^8^ CFU per gram of vaginal fluid in healthy premenopausal women [20, 22].

Our review of the currently available data showed that vaginal *Lactobacillus* suppositories can help to reduce rUTI in both pre- and postmenopausal women. This is interesting given that the vaginal microbiota and causative organisms of rUTI can be different among these two hormonally different groups. Sequencing studies have shown that *L. crispatus* is more abundant in premenopausal than in postmenopausal women, and that *Escherichia coli* is the predominant cause of rUTI in premenopausal females versus a more diverse variety of organisms in postmenopausal women [23]. Despite these differences, however, supplementation with *Lactobacillus* may continue to provide beneficial effects. In our review, studies that included both pre- and postmenopausal women did not separately analyze differences in rUTI in the two cohorts. This would be interesting to further evaluate in future studies.

A significant reduction in rUTI was demonstrated in patients who used the *Lactobacillus* probiotic for 1 year in two prospective studies. These results were not seen in the randomized controlled trials. Additionally, there is no published literature discussing adequate dosing time of probiotics (oral or vaginal) for the prevention of bladder infections. We can only assume that the use must be continuous, as in any other prophylactic regimen such as with antibiotics, and tapered with time, if appropriate.

This review is limited by the available published data as there are few, prospective studies evaluating this topic, in a small total number of patients. Also, there is heterogeneity in the lactobacilli species used among these studies and follow-up time is limited. There remains a paucity of high-quality evidence on this topic, with a continued need for future studies to focus on specific strains of *Lactobacillus*, standardized doses, and larger cohorts of women.

## Conclusion

*Lactobacillus* vaginal probiotics can be used for the prevention of rUTIs with minimal risk. Whether or not they reduce rUTIs in susceptible women remains inconclusive. The appropriate dosing and duration of therapy remain unknown, although our findings demonstrated that continued usage for a longer period of time has more promising results.

## Abbreviation

rUTI: Recurrent urinary tract infection

## References

[1] Recurrent Uncomplicated Urinary Tract Infections in Women: AUA/CUA/SUFU Guideline. 10.1097/JU.0000000000000296. 10.1097/JU.0000000000000296 31042112

[2] Foxman B (2003). Epidemiology of urinary tract infections: incidence, morbidity, and economic costs. Dis Mon.

[3] Foxman B (2014). Urinary tract infection syndromes: occurrence, recurrence, bacteriology, risk factors, and disease burden. Infect Dis Clin North Am.

[4] Gaitonde S, Malik RD, Zimmern PE (2019). Financial burden of recurrent urinary tract infections in women: a time-driven activity-based cost analysis. Urology.

[5] Forssten SD, Sindelar CW, Ouwehand AC (2011). Probiotics from an industrial perspective. Anaerobe.

[6] Borges S, Silva J, Teixeira P (2014). The role of lactobacilli and probiotics in maintaining vaginal health. Arch Gynecol Obstet.

[7] Tachedjian G, Aldunate M, Bradshaw CS, Cone RA (2017). The role of lactic acid production by probiotic Lactobacillus species in vaginal health. Res Microbiol.

[8] Van de Wijgert J, Verwijs M (2020). Lactobacilli-containing vaginal probiotics to cure or prevent bacterial or fungal vaginal dysbiosis: a systematic review and recommendations for future trial designs. BJOG.

[9] Nader-Macías MEF, De Gregorio PR, Silva JA. Probiotic lactobacilli in formulas and hygiene products for the health of the urogenital tract. Pharmacol Res Perspect. 2021;9(5):e00787. 10.1002/prp2.787. 10.1002/prp2.787 PMC8491456 34609059

[10] Baerheim A, Larsen E, Digranes A. Vaginal application of lactobacilli in the prophylaxis of recurrent lower urinary tract infection in women. Scand J Prim Health Care. 1994;12(4):239–43. 10.3109/02813439409029247. 10.3109/02813439409029247 7863140

[11] Bruce AW, Reid G (1988). Intravaginal instillation of lactobacilli for prevention of recurrent urinary tract infections. Can J Microbiol.

[12] Cianci A, Cicinelli E, De Leo V (2018). Observational prospective study on Lactobacillus plantarum P 17630 in the prevention of vaginal infections, during and after systemic antibiotic therapy or in women with recurrent vaginal or genitourinary infections. J Obstet Gynaecol.

[13] Czaja CA, Stapleton AE, Yarova-Yarovaya Y, Stamm WE (2007). Phase I trial of a Lactobacillus crispatus vaginal suppository for prevention of recurrent urinary tract infection in women. Infect Dis Obstet Gynecol.

[14] Reid G, Bruce AW, Taylor M (1992). Influence of three-day antimicrobial therapy and lactobacillus vaginal suppositories on recurrence of urinary tract infections. Clin Ther.

[15] Sadahira T, Wada K, Araki M, Mitsuhata R, Yamamoto M (2021). Efficacy of Lactobacillus vaginal suppositories for the prevention of recurrent cystitis: a phase II clinical trial. Int J Urol.

[16] Stapleton AE, Au-Yeung M, Hooton TM (2011). Randomized, placebo-controlled phase 2 trial of a Lactobacillus crispatus probiotic given intravaginally for prevention of recurrent urinary tract infection. Clin Infect Dis.

[17] Uehara S, Monden K, Nomoto K, Seno Y, Kariyama R, Kumon H (2006). A pilot study evaluating the safety and effectiveness of Lactobacillus vaginal suppositories in patients with recurrent urinary tract infection. Int J Antimicrob Agents.

[18] Scott VCS, Thum LW, Sadun T (2021). Fear and frustration among women with recurrent urinary tract infections: findings from patient focus groups. J Urol.

[19] Gbinigie OA, Tonkin-Crine S, Butler CC, Heneghan CJ, Boylan AM (2022). Non-antibiotic treatment of acute urinary tract infection in primary care: a qualitative study. Br J Gen Pract.

[20] McGroarty JA (1993). Probiotic use of lactobacilli in the human female urogenital tract. FEMS Immunol Med Microbiol.

[21] Van de Wijgert JHHM, Borgdorff H, Verhelst R (2014). The vaginal microbiota: what have we learned after a decade of molecular characterization?. PLoS One.

[22] Farage MA, Miller KW, Sobel JD (2010). Dynamics of the vaginal ecosystem—hormonal influences. Infect Dis (Auckl).

[23] Hugenholtz F, van der Veer C, Terpstra ML (2022). Urine and vaginal microbiota compositions of postmenopausal and premenopausal women differ regardless of recurrent urinary tract infection and renal transplant status. Sci Rep.

